# Molecular Targets and Mechanisms of *Scutellariae radix*-*Coptidis rhizoma* Drug Pair for the Treatment of Ulcerative Colitis Based on Network Pharmacology and Molecular Docking

**DOI:** 10.1155/2021/9929093

**Published:** 2021-06-04

**Authors:** Kai Niu, Qifang Li, Yuan Liu, Yi Qiao, Bingbing Li, Chao Wei, Kunrui Wang, Lu'an Cui, Canlei Zheng, Rong Wang, Li Zhang, Honghua Zhang, Bing Sun, Bin Yu

**Affiliations:** ^1^College of Integrated Chinese and Western Medicine, Jining Medical University, Jining 272067, China; ^2^Department of Traditional Chinese Medicine, Affiliated Hospital of Jining Medical University, Jining 272060, China; ^3^School of Public Health, Jining Medical University, Jining 272067, China; ^4^College of Pharmaceutical Sciences, Hangzhou Normal University, Hangzhou 311121, China

## Abstract

This study aims to analyze the targets of the effective active ingredients of *Scutellariae radix*-*Coptidis rhizoma* drug pair (SCDP) in ulcerative colitis (UC) by network pharmacology and molecular docking and to explore the associated therapeutic mechanism. The effective active ingredients and targets of SCDP were determined from the TCMSP database, and the drug ingredient-target network was constructed using the Cytoscape software. The disease targets related to UC were searched in GeneCards, DisGeNET, OMIM, and DrugBank databases. Then, the drug ingredient and disease targets were intersected to construct a protein-protein interaction network through the STRING database. The Metascape database was used for the Gene Ontology and Kyoto Encyclopedia of Genes and Genomes pathway enrichment analyses of the predicted targets of SCDP for UC. The Autodock software was used for molecular docking between the main active ingredient and the core target to evaluate the binding ability. SCDP has 43 effective active ingredients and 134 intersection targets. Core targets included AKT1, TP53, IL-6, VEGFA, CASP3, JUN, TNF, MYC, EGFR, and PTGS2. GO functional enrichment analysis showed that biological process was mainly associated with a cytokine-mediated signaling pathway, response to an inorganic substance, response to a toxic substance, response to lipopolysaccharide, reactive oxygen species metabolic process, positive regulation of cell death, apoptotic signaling pathway, and response to wounding. KEGG enrichment analysis showed main pathway concentrations were related to pathways in cancer, AGE-RAGE signaling pathway in diabetic complications, bladder cancer, IL-17 signaling pathway, apoptosis, p53 signaling pathway, and PI3K-Akt signaling pathway. The drug active ingredient-core target-key pathway network contains 41 nodes and 108 edges, of which quercetin, wogonin, baicalein, acacetin, oroxylin A, and beta-sitosterol are important active ingredients; PTGS2, CASP3, TP53, IL-6, TNF, and AKT1 are important targets; and the pathways involved in UC treatment include pathways in cancer, PI3K-Akt signaling pathway, AGE-RAGE signaling pathway in diabetic, apoptosis, IL-17 signaling pathway and herpes simplex infection. The active ingredient has a good binding capacity to the core target. SCDP key active ingredients are mainly quercetin, wogonin, baicalein, acacetin, oroxylin A, and beta-sitosterol, which function mainly by regulating targets, such as PTGS2, CASP3, TP53, IL-6, TNF, and AKT1, and are associated with multiple signaling pathways as pathways in cancer, PI3K-Akt signaling pathway, apoptosis, IL-17 signaling pathways.

## 1. Introduction

Ulcerative colitis (UC), a type of inflammatory bowel disease (IBD), is a chronic inflammatory disease of the intestine characterized by abnormal intestinal mucosal structure, changes in intestinal bacterial composition, and systemic biochemical dysfunction. It is an incurable disease with low mortality [1]. The incidence of this disease is 11.60 per 100,000 individuals and 24.5 per 100,000 individuals in China and Hong Kong, respectively, which is lower than the incidence in Western countries but shows an increasing annual trend [2–5]. The main clinical symptoms are abdominal pain, diarrhea, bloody purulent stools, and weight loss accompanied by numerous neutrophils, macrophages, and inflammatory factors infiltrating the intestinal mucosa. The pathogenesis of this disease is not clear, and studies suggest a complex interaction among host genetic factors, immunity, and microbial environmental exposure [6]. In addition to aminosalicylic acid preparations and corticosteroids, immunomodulators, Janus kinase inhibitors, and some biological agents, such as tumor necrosis factor-alpha (TNF-*α*), interleukin (IL)-17A, and IL-12/IL-23p40 antibodies, have been applied in the clinical treatment of UC [7]. However, more and more patients eventually become refractory or intolerant to the side effects or complications of drugs, and the persistence of chronic inflammatory conditions often becomes a risk factor for inducing colorectal cancer [8, 9]. Under the circumstance, traditional Chinese medicines (TCMs) play an important role in the treatment of UC and are regarded as complementary and alternative medicine treatment options. Also, much evidence has shown that TCM therapies, including Chinese herbal medicine, Chinese patent medicine, acupuncture, moxibustion, have potentially positive effects on UC [10, 11].

*Scutellariae radix-Coptidis rhizoma* drug pair is the main ingredient herb for clearing away heat and dampness. Their compatibility is often used to treat damp-heat syndromes, dysentery, diarrhea, and other diseases [12–14]. Furthermore, they are the main ingredient drugs in the classical prescriptions as Gegen Qinlian decoction, Wumei pill, Banxia Xiexin decoction, and Shaoyao decoction, and others [15–18]. The combination of *Scutellaria baicalensis* and *Coptis chinensis* is mainly responsible for clearing away heat and dampness and has an antidiarrheal and antidysentery efficacy. Furthermore, Hu et al. used a database to analyze the medication regularity of TCM compounds in the treatment of UC. Results showed that *Scutellaria baicalensis* and *Coptis chinensis* were among the top 26 drugs with the highest medication, of which and *Coptis chinensis* was the most frequently used drug [19]. Professor Jingri Xie advocated that *Scutellaria baicalensis*, *Coptis chinensis*, *Phellodendron amurense*, and *Sophora flavescens* can be used together and they are particularly suitable for patients with the damp-heat syndrome, such as abdominal bloat, bitter mouth, nausea, vomiting, and/or yellow tongue coating [20]. Previous work in our laboratory also confirmed that *Scutellaria baicalensis* and *Coptis chinensis* are the drugs which were used more frequently in the treatment of UC and are commonly paired drugs to treat damp-heat intrinsic syndrome [21, 22].

One proposed mechanism of the *Scutellariae radix-Coptidis rhizoma* drug pair (SCDP) in the treatment of UC is that baicalein can reduce the number of apoptotic intestinal epithelial cells due to its ability to reduce the expression of the 78-kDa glucose-regulated protein (GRP78) and caspase-3 [23]. Researchers have found that berberine-nanostructured lipid carriers can block the nuclear translocation of nuclear factor-kappa B (NF-*κ*B), reduce the expression of the proinflammatory cytokines IL-1*β*, IL-6, matrix metalloproteinase-9, chemokine motif receptor 1 (CX3CR1), cyclooxygenase-2 (COX-2), and telomerase reverse transcriptase, and increase the expression of the tight junction scaffolding protein zonula occludens-1, to play a therapeutic role in UC [24]. Gegen Qinlian decoction, comprising herbs *Scutellariae radix* and *Coptidis rhizoma*, exert therapeutic effects by inhibiting the expression of toll-like receptor 4 (TLR4)/NF-*κ*B pathway and suppressing the inflammatory factors secretion as TNF-*α*, IL-6, IL-1*β*, and IL-4 in the colon of UC animal models [15]. In addition, a recent report has revealed that baicalin, which was the main ingredient of *Coptidis rhizoma*, can exert a therapeutic effect on UC through increasing glutathione peroxidase and superoxide dismutase contents and inhibiting oxidative stress response as well as apoptosis [10].

Network pharmacology and molecular docking are new technologies based on systems biology and databased molecular correlation analysis in the exploration of new drugs and prediction of drug targets. Network pharmacology emphasizes multi-ingredient-multitargets-multipathway regulation of the signal pathway. Many studies have confirmed that network pharmacology is significant for determining the target of drugs and screening the active ingredients of drugs [25–27]. In this study, the mechanism of *Scutellariae radix-Coptidis rhizoma* drug pair in the treatment of UC was analyzed by network pharmacology technology and molecular docking approaches to help better guide clinical treatment and mechanism research.

## 2. Materials and Methods

### 2.1. SCDP Ingredients Collection and Target Gene Prediction

The Traditional Chinese Medicine Systems Pharmacology (TCMSP) database and analysis platform (https://tcmspw.com/tcmsp.php) was used for the active ingredient screening. Oral bioavailability (OB) ≥30% and drug-likeness (DL) ≥0.18 were used as absorption, distribution, metabolism, and excretion parameters. Target genes were predicted using TCMSP and the Swiss Target Prediction database (http://www.swisstargetprediction.ch/) after identifying the active ingredients. The names of the predicted target genes were standardized from the UniProt database (https://www.uniprot.org/). The SCDP ingredients and target gene network were drawn using the Cytoscape software, and the top 10 active ingredients were identified by their degree values.

### 2.2. Prediction of UC-Related Pathogenic Genes

GeneCards (https://www.genecards.org/), DisGeNET (https://www.disgenet.org/), OMIM (http://www.omim.org/), and DrugBank (https://www.drugbank.ca/) were selected to obtain UC-related pathogenic genes. Both databases can be used to find the most cutting-edge disease-related genes. All the databases were used “ulcerative colitis” as the keyword.

### 2.3. Protein-Protein Interaction (PPI) Analysis and Hub Genes

PPI is particularly important for biological process analyses and is vital for understanding complex mechanisms in a living cell. PPI network mapping was performed on the obtained bioactive ingredients and disease targets using the Search Tool for the Retrieval of Interacting Genes (STRING) database (http://string-db.org/) with the species limited to “*Homo sapiens*” and a confidence score of >0.4. Next, the interaction files were downloaded and imported into Cytoscape to visualize the PPI network and analyze the topological value. Then, based on the MCODE plug-in, the targets with a median value and a median value that was not less than the average value were recognized as the key targets of SCDP for the treatment of UC. And the top 10 ranked proteins were defined as hub targets based on the degree level.

### 2.4. Enrichment Analysis of Gene Ontology (GO) and Kyoto Encyclopedia of Genes and Genomes (KEGG)

The predicted targets were imported into the Metascape database (http://metascape.org/) for GO and KEGG enrichment analyses by entering the list of intersection target gene names and selecting the species as “*Homo sapiens*” for customized analysis with a cutoff *P* value of <0.01. According to the results, a network visualization was constructed by the tools in the bioinformatics website (http://www.bioinformatics.com.cn).

### 2.5. SCDP Drug Ingredient-Target Genes-Pathway Interaction Network

The SCDP active ingredients predicted target genes, and the top 20 key pathways for enrichment analysis were imported into the Cytoscape software to construct the drug ingredient-target genes-pathway interaction network. Topological analysis was performed on the constructed network according to the values of degree centrality, betweenness centrality, and closeness centrality.

### 2.6. Molecular Docking

Molecular docking is used to interpret the binding area of small molecule ligands and macromolecular receptors through computer simulation and then calculates the physical and chemical parameters for predicting the affinity between the two. Receptor and ligand files were downloaded from PubChem (https://pubchem.ncbi.nlm.nih.gov/) and PDB (https://www.rcsb.org) databases.

And then, molecular docking was performed using the AutoDockTools software. The docking range between the protein and the active ingredient was set using AutoGrid, and molecular docking was performed by running the “Genetic Algorithm” using Docking settings, with binding energy less than 0 indicating a good potential for ligand binding to the receptor.

## 3. Results

### 3.1. SCDP Ingredients Collection and Target Gene Prediction

According to the two screening conditions of OB value and DL index, 50 active ingredients of SCDP, including 36 ingredients in *Scutellariae radix* and 14 ingredients in *Coptidis rhizoma,* were obtained from TCMSP. Eight ingredients were eliminated because they showed no predicted targets in either the TCMSP or Swiss Target Prediction databases. Finally, 43 ingredients, including 32 in *Scutellariae radix*, 11 in *Coptidis rhizoma*, and coptisine and epiberberine as two common ingredients, were obtained (Table 1). Then the target genes of 43 active ingredients were collected for target genes prediction in TCMSP and Swiss Target Prediction. After merging the data and deleting duplicates, 223 target genes were finally distinguished.

### 3.2. Drug Active Ingredient-Target Gene Interaction Network

**Figure 1:**
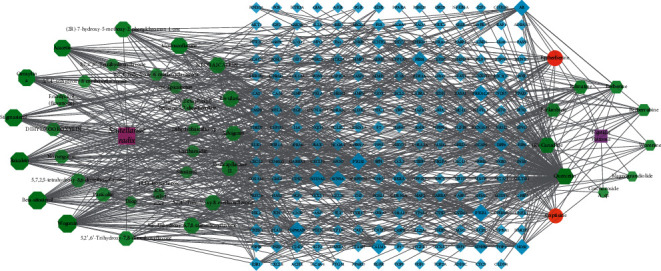
Drug ingredient-target interaction network. The purple squares represent the drug Scutellariae radix and Coptidis rhizoma. The green octagons represent the ingredients in Scutellariae radix, while green hexagons represent the ingredients of Coptidis rhizoma. The red circle represents the common active ingredient of SCDP. The blue diamond represents the predicted target. The area and color transparency of the node represent its value. The darker the area and color, the more important the node. SCDP, Scutellariae radix-Coptidis rhizoma drug pair.

Drug active ingredient-target gene interaction networks were obtained by importing the active ingredients and predicted genes into Cytoscape 3.8.0. There are 266 nodes and 824 edges (Figure 1). The edges represent the interrelationships between the drug and active ingredients and between the ingredients and targets. Meanwhile, the degree value represents the number of routers connected to each node in the network. The higher the degree value, the more likely the node plays a key role in the network. In this network, the same compound acted on multiple targets. Simultaneously, multiple compounds shared the same target. Furthermore, the top 10 key active ingredients and 19 core targets (degree ≥10) were screened according to the degree value as shown in Tables 2 and 3.

The purple squares represent the drug *Scutellariae radix* and *Coptidis rhizoma*. The green octagons represent the ingredients in *Scutellariae radix*, while green hexagons represent the ingredients of *Coptidis rhizoma*. The red circle represents the common active ingredient of SCDP. The blue diamond represents the predicted target. The area and color transparency of the node represent its value. The darker the area and color, the more important the node. SCDP: *Scutellariae radix-Coptidis rhizoma* drug pair.

### 3.3. Prediction of UC-Related Pathogenic Genes

The disease genes related to UC were collected from GeneCards, DisGeNET, OMIM, and DrugBank, with “*ulcerative colitis*” as the keyword. Taking 1.795 and 4.51 as the median “Relevance score,” 1111 relevant targets were screened in GeneCards. Meanwhile, 1458, 552, and 65 pathogenic genes were obtained from the DisGeNET, OMIM, and DrugBank databases, respectively. The UniProt database was used to standardize target names, and finally a total of 2386 UC-related pathogenic target genes were obtained after collating and removing duplicates.

### 3.4. Construction of the PPI Network and Core Target Screening

**Figure 2:**
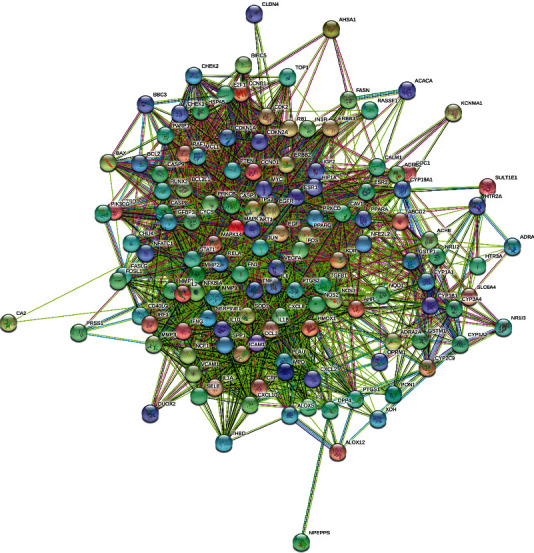
Common targets in the PPI network of SCDP for the treatment of ulcerative colitis. The PPI network includes 134 nodes, 2612 edges, and an average node degree of 39. PPI: protein-protein interaction; SCDP: *Scutellariae radix-Coptidis rhizoma* drug pair.

A total of 134 intersection targets were obtained by screening drug ingredient targets and disease targets using the Wayne diagram. The PPI network was construed by the STRING databases of the 134 targets, as shown in Figure 2, with 134 nodes, 2612 edges, an average node degree of 39, and a PPI enrichment *p* < 1.0*e* − 16.

**Figure 3:**
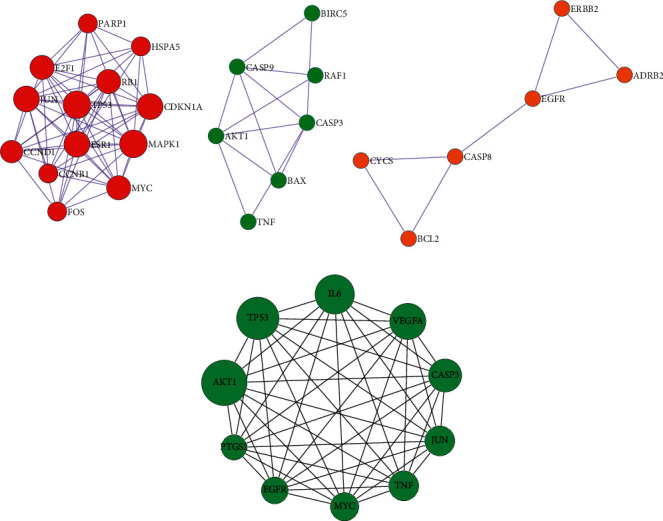
The core target PPI network module clusters (a) and core protein network diagram of SCDP for the treatment of ulcerative colitis (b). PPI: protein-protein interaction; SCDP: *Scutellariae radix-Coptidis rhizoma* drug pair.

The obtained TSV format files of the PPI network were imported into the Cytoscape software for visualization analysis. As the interactions between proteins in the PPI networks are reciprocal, it is usually classified as an undirected graph. Some regions with high density were present in the PPI complex network, and they are called a community or module. The networks within modules are potential subnetworks of the PPI network, with a high density of connections in the subnetworks and few connections in other regions. Therefore, modules are considered to be biologically significant sets. The sets have two meanings: (1) protein complex, (i.e., multiple proteins combine to form a complex, which then plays a biological role); (2) functional module (e.g., proteins located in the same pathway interact more closely). The MCODE plug-in was then applied to analyze the interactions using a molecular complex detection algorithm, and 3 module clusters were obtained (Figure 3). According to the degree values, the top 10 core targets were identified as AKT1, TP53, IL-6, VEGFA, CASP3, JUN, TNF, MYC, EGFR, and PTGS2 (Figure 3), whose full names are provided in Table 4.

### 3.5. GO and KEGG Enrichment Analyses

**Figure 4:**
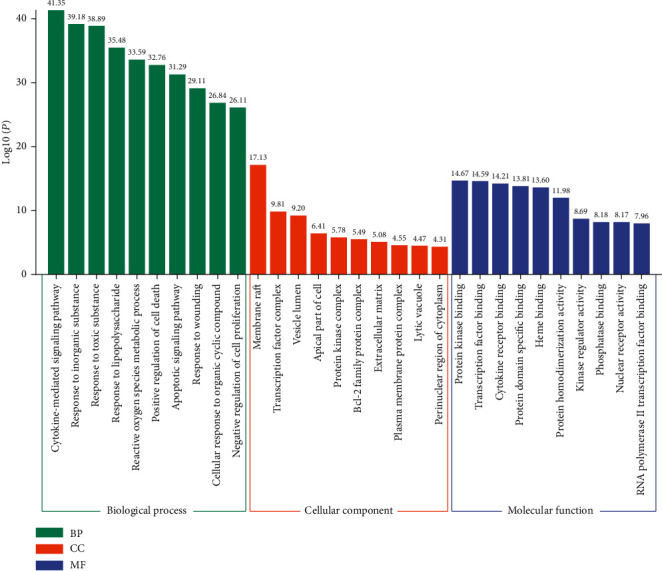
GO terms of candidate targets of SCDP against ulcerative colitis. The top 10 GO functional categories were selected. GO: gene ontology; SCDP: *Scutellariae radix-Coptidis rhizoma* drug pair; BP: biological process; CC: cellular component; MF: molecular function.

Metascape was used for GO and KEGG analyses of the 134 hub genes to obtain enriched ontology clusters. As a result, we obtained 2304 GO terms, including 2073 biological processes (BPs), 79 cellular components (CCs), and 152 molecular functions (MFs).  Figure 4 shows that BPs were mainly related to cytokine-mediated signaling pathway, response to inorganic substances, response to toxic substances, response to lipopolysaccharides (LPS), reactive oxygen species (ROS) metabolic processes, positive regulation of cell death, apoptotic signaling pathway, and response to wounding. The CCs were mainly related to membrane raft and transcription factor complex. Moreover, the MFs were mainly related to protein kinase binding, transcription factor binding, cytokine receptor binding, and protein homodimerization activity (Figure 4).

**Figure 5:**
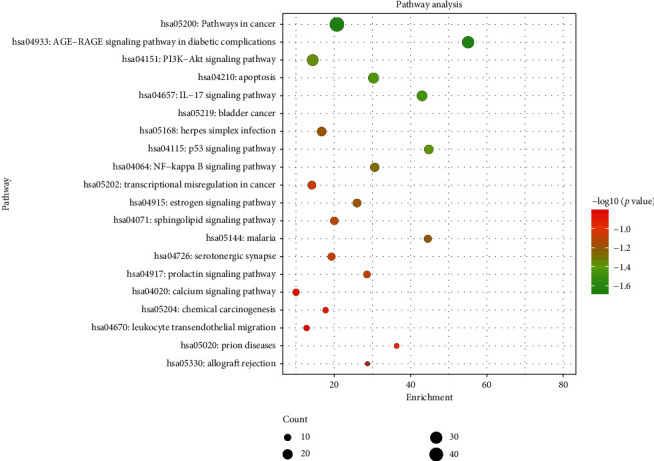
KEGG pathway enrichment of top 20 pathways of SCDP in the treatment of ulcerative colitis. The size of the spot represents the number of genes, and the color represents enrichment of -log10 (*p* value). KEGG: Kyoto Encyclopedia of Genes and Genomes; SCDP: *Scutellariae radix-Coptidis rhizoma* drug pair.

KEGG pathway enrichment analysis screening resulted in 164 signaling pathways. Visual analysis of the top 20 pathways showed that the main signaling pathways of SCDP in the treatment of UC were concentrated in pathways in cancer, the advanced glycation end products and the receptor for AGEs (AGE-RAGE) signaling pathway in diabetic complications, bladder cancer, IL-17 signaling pathway, apoptosis, p53 signaling pathway, and phosphoinositide 3-kinase (PI3K)-Akt signaling pathway (Figure 5).

### 3.6. Drug Ingredient-Target-Pathway Interaction Network Construction

**Figure 6:**
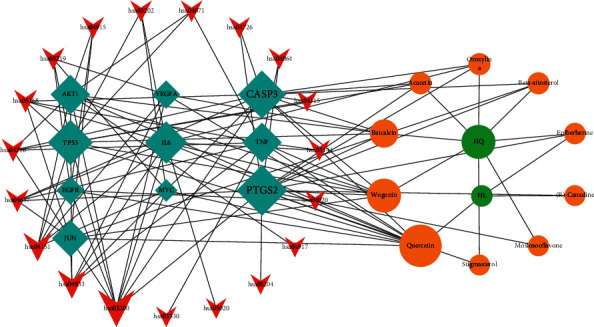
Key ingredient-protein target-pathway network for SCDP in the treatment of ulcerative colitis. Green circles represent the *Scutellariae radix* as “HQ” and *Coptidis rhizoma* as “HL.” Orange circle represents key active ingredients, while blue diamond represents key protein targets. The red V-shape represents key pathways from KEGG enrichment analysis. The area and color transparency of the node represent its degree; the darker the area and color, the more important the node. SCDP: *Scutellariae radix-Coptidis rhizoma* drug pair.

The Cytoscape software was used to construct an active ingredient-core target-key pathway network, which contained 41 nodes and 108 edges (Figure 6). According to the degree values (betweenness centralities and closeness centralities in the drug ingredient-target-pathway network), the main active ingredients of SCDP for UC treatment were determined to be quercetin, wogonin, baicalein, acacetin, oroxylin A, and beta-sitosterol, with the main targets being PTGS2, CASP3, TP53, IL-6, TNF, and AKT1 (Tables 5 and 6).

### 3.7. Molecular Docking

**Figure 7:**
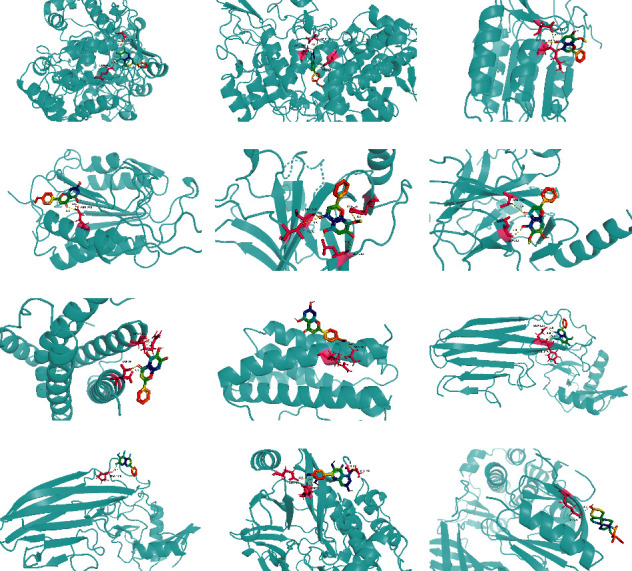
Docking results of the key ingredient and six hubs target proteins. (a) Baicalein and PTGS2; (b) acacetin and PTGS2; (c) wogonin and CASP3; (d) acacetin and CASP3; (e) wogonin and TP53; (f) oroxylin A and TP53; (g) baicalein and IL-6; (h) acacetin and IL-6; (i) wogonin and TNF; (j) baicalein and TNF; (k) quercetin and AKT1; (l) beta-sitosterol and AKT1.

The molecular docking results indicate binding energies of less than −2 kcal/mol after docking, suggesting the formation of stable bonds between the active ingredients and predicted target proteins (Table 7). The PyMOL software was used to visualize the docking results of key active ingredients and core targets, as shown in Figure 7.

## 4. Discussion

UC is a type of irritable bowel syndrome (IBS), and the main pathogenesis of UC is spleen deficiency as the original cause and damp-heat with stasis toxin as the superficial factors in TCM theory [28–30]. Based on the theory of TCM, UC consists of several types of syndromes, the most common of which (34.8%) being damp-heat accumulation syndrome [4, 31]. Therefore, the main treatment approach is to enhance the spleen Qi and eliminate damp-heat toxin. Damp-heat and stasis toxin are important factors in the recurrence of this disease [32].

SCDP is a commonly used drug pair for eliminating damp-heat toxin. These two herbs are also constituents of many classical formulas, such as decoctions Gegen Qinlian, Huangqin, Shaoyao, Baitouweng, Huanglian Jiedu, Banxia Xiexin, and WuMei pill, for the treatment of diarrhea as a symptom of UC, by TCMs [27].

The damp-heat insidious pathogen has been proposed as the mechanism responsible for UC recurrence, necessitating that damp-heat toxin is cleared for UC treatment. In turn, Cao et al. advocated the use of *Scutellariae radix*, *Coptidis rhizoma*, *Cortex Phellodendri*, *Sophora flavescens*, *pulsatilla*, *ash bark*, among others, in combination with dampness-dissipating drugs, such as *Pogostemon cablin* and *eupatorium*, while Y. Zhang et al. suggested the use of *Coptidis rhizoma*, *Scutellariae radix*, *Cortex Phellodendri, Sophora flavescens,* and *pulsatilla* as the main ingredients in the formulation [27, 33, 34]. To further clarify the internal mechanism of SCDP for UC treatment, we applied network pharmacology to analyze its active ingredients, targets, and pathways, thus providing ideas for future research and drug development.

The results show that the key active ingredients are quercetin, wogonin, baicalein, acacetin, oroxylin A, and beta-sitosterol. Previous research discovered that quercetin inhibits the expression of LPS-induced inflammatory genes, mainly by reducing the levels of TNF-*α* and lipocalin-2 mRNA and enhancing the expression of the Slip protein to protect the UC mucosa [35]. It has been found that quercetin exerts a therapeutic effect in IBD by regulating the structure of the intestinal flora, inhibiting the expression of proinflammatory factors, such as IL-17, TNF-*α*, IL-6, and increasing the expression of IL-10 [36].

Wogonin has strong anti-inflammatory, antitumor, antiviral and antiallergic properties. It can inhibit the activity of Treg cells, regulate the differentiation of Th17 cells, and inhibit the transcription of the extracellular signal-regulated kinase (ERK), signal transducer and activator of transcription 3 (STAT3), and hypoxia-inducible factor-1*α* to regulate inflammatory responses [37]. One study found that 25-mg/mL wogonin can reduce the LPS-induced inflammatory response of the TLR4-MyD88-mediated NF-*κ*B pathway in Caco-2 cells, suggesting its protective effect on the intestinal mucosal barrier [38].

Baicalein is the main ingredient of *Coptidis rhizoma*. Studies have suggested its anti-inflammatory effect and discovered that it could improve the inflammatory state of UC and the control drugs [39–41]. The effect of baicalein is similar to that of the reference drug sulfasalazine and comparable to wogonin [42, 43]. In addition, baicalein reduces the levels of the inflammatory mediators IL-33 and NF-*κ*B p65 and increases the level of I*κ*B*α* to resists the pathological changes of dextran sulfate sodium (DSS)-induced UC [44].

Acacetin (5,7-dihydroxy-4′-methoxyflavone) is an O-methylated flavone naturally present in plants like chrysanthemum and safflower, as well as in Calamintha and Linaria species. Acacetin possesses antiperoxidative, anti-inflammatory, antiplasmodial, and anticancer activities. In addition, acacetin strongly inhibits the expression of proinflammatory cytokines, inducible nitric oxide synthase (iNOS), and COX-2 in LPS-induced RAW 264.7 cells [45]. Research has shown that acacetin treatment could inhibit sepsis-induced acute lung injury and reduced iNOS and COX-2 expression.

Oroxylin A and baicalein are produced as major metabolites of baicalin by the action of bacteria in the large intestine cavity. In addition, both baicalin and baicalein are converted into four new metabolites: wogonin, wogonoside, oroxylin A, and oroxin A [46]. Some studies have shown that oroxylin A is involved in the induction of autophagy and apoptosis, potentially via its regulation of p62-mediated caspase-8, which promotes the transcription of NF-*κ*B downstream target genes and inhibits the transcription of downstream target genes of Nrf2 [47–49].

Beta-sitosterol was found to markedly reduce weight loss, colonic length shortening, and microscopic changes in DSS-induced colitis and to reduce TNF-*α*, IL-6, and IL-1*β* levels in the intestinal tissues of experimental colitis mice in a concentration-dependent manner [50].

The core targets of SCDP in UC treatment are PTGS2 (COX2), CASP3, TP53, IL-6, TNF, and AKT1, which are associated with the TNF signaling pathway and NF-*κ*B and oxidative stress pathways. An important target of anti-inflammatory drugs, especially nonsteroidal anti-inflammatory drugs, is prostaglandin endoperoxide synthase or COX. There are two subtypes of COX. COX1 is constitutively expressed and is responsible for regulating normal physiological processes. COX2 is induced under inflammatory conditions and mainly regulates the inflammatory process. It has been suggested that the expression of COX2 is induced in the colon of IBD patients but also in the inflamed tissues of IL-10-deficient IBD mouse models, and reduced COX2 is considered a major target for IBD treatment [46].

A previous study in UC rats revealed that baicalin could up-regulate B-cell lymphoma 2 (Bcl-2) protein expression, lower Bax (also known as Bcl-2-like protein 4) protein expression, induce marked activation of the caspase-cascade system, and exhibit concentration-dependent down-regulation of Fas and Fas Ligand (FasL) protein expressions to exert therapeutic effects in UC [10]. AKT1 is a key mediator of the PI3K-Akt signaling pathway and is involved in cellular function regulation in various tumors, including gastric cancer, glioma, lung cancer, and esophageal squamous cell carcinoma [51, 52].

Berberine has been found to play a regulatory role in macrophage M1 polarization in DSS-induced colitis through the AKT1-suppressor of cytokine signaling-1 (SOCS1)-NF-*κ*B signaling pathway [53]. IL-6 gene expression is strongly associated with IBD progression [54]. JUN is a proto-oncogene that plays a key role in inflammation, and can be activated by many inflammatory factors directly or indirectly. The activation of JUN further modulates the expression and regulation of relevant inflammatory factors, which, in turn, participate in the regulation of the inflammatory response [55]. Being a proinflammatory regulator, TNF promotes the release of proinflammatory factors and plays an important role in the development and progression of UC. In addition, together with interferons, it alters the barrier function of intestinal epithelial cells, enhances the permeability of the intestinal mucosa and vascular wall, and disrupts the integrity of the intestinal mucosa, leading to ulcer formation [56, 57].

GO analysis and KEGG enrichment results showed that the mechanism of SCDP in the treatment of UC was mainly related to the following pathways: pathways in cancer, PI3K-Akt signaling pathway, the AGE-RAGE signaling pathway in diabetic complications, apoptosis, IL-17 signaling pathway, and herpes simplex infection.

According to Rogler, up to 30% of the patients with chronic active UC or Crohn's disease (CD) are likely to get colorectal cancer after 35 years since the onset of the disease [58]. Chronic inflammation, the increased turnover of epithelial cells, and ROS production are thought to contribute to the development of dysplastic lesions, which may then transform into colorectal cancer [59]. Meanwhile, chronic UC status is also a major risk factor in the development of colitis-associated cancer [59].

IL-17, mainly secreted by Th17 cells, is involved in inflammatory response, and its levels have been confirmed to correlate with the extent of UC disease. Acting as a proinflammatory factor, IL-23 promotes IL-17 production and forms the IL-23/17 axis to amplify the inflammatory response [60, 61]. IL-17A induces the aggregation of neutrophils on target cells, resulting in the secretion of inflammatory cytokines (IL-6, TNF-*α*, and IL-1B), chemokines (CXC), granulocyte colony-stimulating factor, and granulocyte-macrophage colony-stimulating factor. Meanwhile, IL-17A is also involved in the regulation of mucosal barrier function and amplification of the complete inflammatory response through the production of antimicrobial peptides, such as *β*-defensins, S100 *β*-defensins, and S100 proteins [62]. The strongest evidence comes from the genome-wide association studies that have identified IBD susceptibility single nucleotide polymorphisms in many genes involved in the IL-23/IL-17 axis [63, 64]. Other susceptibility genes in Crohn's disease and UC also appear to be involved in the IL-23/IL-17 axis. For example, STAT3, janus kinase 2 (JAK2) and tyrosine kinase 2 (TYK2) participate in IL-23 signaling, and IL-12B encodes the common subunit of IL-12 and IL-23 [61].

The mutation of the p53 gene is an important genetic alteration involved in the early stages of UC-associated colorectal cancer. The overexpression of p53 protein in the colorectal crypt of UC patients, usually in the absence of atypical hyperplasia observation of atypical hyperplasia, is used by pathologists to define the state between regenerative changes and intraepithelial neoplasia. It has been used as a biomarker in predicting the risk of progression into a malignant tumor. High-frequency mutations of p53 were reported in severe chronic UC patients with undiagnosed cancer [65]. The expression of p53 in UC patients showed a negative correlation with IL-6, which is mainly involved in the development of cancer-induced by chronic inflammation. IL-6 is the main activator of STAT3, and its stimulation products increase the transcription of target genes, as well as cell proliferation/survival. Downregulation of p53 expression is probably the most important step in the transformation of inflamed tissues and cells. Particularly, inflammatory cells that stimulate proliferation and inhibit apoptosis of cells, cytokines, chemokines, and growth factors produce ROS and reactive nitrogen species that cause oxidative DNA damage. The damage may not be adequately repaired because the p53 function is downregulated by IL-6 [66].

In addition, TCMs may exert relevant apoptotic inhibitory effects through the upregulation of p53 and Bcl-2 expression, which is accompanied by the downregulation of TNF-*α* and IL-1 and the upregulation of IL-6 and IL-10. The PI3K-AKT signaling pathway is involved in the regulation and release of proinflammatory cytokines, including TNF-*α* and other cytokines closely related to the inflammatory response of UC [67], and plays an important role in the development and progression of UC. TCMs inhibit transforming growth factor-*β* (TGF-*β*) expression and lead to a decrease in PI3K/AKT activation, thereby alleviating the symptoms of UC by regulating TGF-*β* expression [68]. AGE and RAGE are involved in and mediate various signaling pathways of oxidative stress, induce ROS production, and activate NF-*κ*B, leading to inflammatory responses, cell apoptosis, and microvascular diseases [69]. RAGE polymorphisms and increased RAGE levels have been found in IBD patients, and the involvement of AGE/RAGE in inflammation correlates with its activation of NF-*κ*B and its response to oxidative stress [70].

The results of molecular docking showed that quercetin, wogonin, baicalein, acacetin, oroxylin A, and beta-sitosterol, the main active ingredients of SCDP, had a good affinity to the core target genes *PTGS2*, *CASP3*, *TP53*, *IL-6*, *TNF*, and *AKT1*, suggesting that the key active ingredients of SCDP play a therapeutic role in UC by mainly intervening with the key signaling targets, such as the inflammatory response and oxidative stress. In summary, the potential biological mechanism of SCDP in the treatment of UC involves multiple ingredients, targets, and pathways, and the therapeutic effects occur through several pathways as pathways in cancer, PI3K-Akt signaling pathway, apoptosis, and IL-17 signaling pathway.

The presented study provides a scientific basis for the subsequent development and utilization of SCDP. Even so, network pharmacology has only analyzed the main active ingredients and targets of drugs, and further validation of their predicted target pathways is needed experimentally. The conclusions of this research also contribute to the research and development of drugs and further elaboration of mechanisms.

## Figures and Tables

**Table 1:** Ingredients in *Scutellariae radix*-*Coptidis rhizoma* drug pair.

Drug source	Molecule ID	Molecule name	OB (%)	DL
*Scutellariae radix*	MOL001689	Acacetin	34.97	0.24
	MOL000173	Wogonin	30.68	0.23
	MOL000228	(2*R*)-7-Hydroxy-5-methoxy-2-phenylchroman-4-one	55.23	0.2
	MOL002714	Baicalein	33.52	0.21
	MOL002909	5,7,2,5-Tetrahydroxy-8,6-dimethoxyflavone	33.82	0.45
	MOL002910	Carthamidin	41.15	0.24
	MOL002913	Dihydrobaicalin_qt	40.04	0.21
	MOL002914	Eriodyctiol (flavanone)	41.35	0.24
	MOL002915	Salvigenin	49.07	0.33
	MOL002917	5,2′,6′-Trihydroxy-7,8-dimethoxyflavone	45.05	0.33
	MOL002925	5,7,2′,6′-Tetrahydroxyflavone	37.01	0.24
	MOL002927	Skullcapflavone II	69.51	0.44
	MOL002928	Oroxylin A	41.37	0.23
	MOL002932	Panicolin	76.26	0.29
	MOL002933	5,7,4′-Trihydroxy-8-methoxyflavone	36.56	0.27
	MOL002934	Neobaicalein	104.34	0.44
	MOL002937	Dihydrooroxylin	66.06	0.23
	MOL000358	Beta-sitosterol	36.91	0.75
	MOL000359	Sitosterol	36.91	0.75
	MOL000525	Norwogonin	39.4	0.21
	MOL000552	5,2′-Dihydroxy-6,7,8-trimethoxyflavone	31.71	0.35
	MOL000073	Ent-epicatechin	48.96	0.24
	MOL000449	Stigmasterol	43.83	0.76
	MOL001458	Coptisine	30.67	0.86
	MOL001490	Bis[(2*S*)-2-ethylhexyl] benzene-1,2-dicarboxylate	43.59	0.35
	MOL002879	Diop	43.59	0.39
	MOL002897	Epiberberine	43.09	0.78
	MOL008206	Moslosooflavone	44.09	0.25
	MOL010415	11,13-Eicosadienoic acid, methyl ester	39.28	0.23
	MOL012245	5,7,4′-Trihydroxy-6-methoxyflavanone	36.63	0.27
	MOL012246	5,7,4′-Trihydroxy-8-methoxyflavanone	74.24	0.26
	MOL012266	Rivularin	37.94	0.37

*Coptidis rhizoma*	MOL001454	Berberine	36.86	0.78
	MOL002894	Berberrubine	35.74	0.73
	MOL002897	Epiberberine	43.09	0.78
	MOL002903	(*R*)-Canadine	55.37	0.77
	MOL002904	Berlambine	36.68	0.82
	MOL002907	Corchoroside A_qt	104.95	0.78
	MOL000622	Magnograndiolide	63.71	0.19
	MOL000785	Palmatine	64.6	0.65
	MOL000098	Quercetin	46.43	0.28
	MOL001458	Coptisine	30.67	0.86
	MOL002668	Worenine	45.83	0.87

OB: oral bioavailability; DL: drug-likeness.

**Table 2:** Top 10 key active ingredients in *Scutellariae radix*-*Coptidis rhizoma* drug pair.

Rank	Molecule ID	Name	Degree ≥24
1	MOL000098	Quercetin	151
2	MOL000173	Wogonin	46
3	MOL000358	Beta-sitosterol	38
4	MOL002714	Baicalein	38
5	MOL000449	Stigmasterol	32
6	MOL002903	(R)-Canadine	32
6	MOL002928	Oroxylin A	27
8	MOL001689	Acacetin	27
9	MOL008206	Moslosooflavone	25
10	MOL002897	Epiberberine	24

**Table 3:** Core targets of active ingredients in the *Scutellariae radix-Coptidis rhizoma* drug pair.

Rank	Name	Degree ≥ 10	Closeness centrality	Neighbourhood connectivity
1	PTGS2	37	0.5248	22.3429
2	PTGS1	33	0.5166	22.9375
3	HSP90AB1	30	0.5009	23.4667
4	PRKACA	26	0.5009	24.5200
5	AR	25	0.4514	25.5652
6	SCN5A	24	0.4775	26.5652
7	NCOA2	24	0.4809	24.7391
8	PRSS1	23	0.4484	27.5238
9	NOS2	23	0.3228	19.6190
10	CALM1	22	0.3529	21.5909
11	DPP4	17	0.4368	29.4706
12	RXRA	16	0.4530	31.4667
13	ADRB2	15	0.4625	32.8000
14	KCNH2	15	0.4366	30.9231
15	PIK3CG	14	0.4515	32.4286
16	NOS3	14	0.3973	29.8182
17	ESR1	13	0.3078	18.1818
18	F10	11	0.4213	32.2727
19	NCOA1	10	0.3236	24.0000

**Table 4:** The main targets of *Scutellariae radix*-*Coptidis rhizoma* drug pair.

Rank	Gene symbol	Gene name	Degree
1	AKT1	RAC-alpha serine/threonine-protein kinase	108
2	TP53	Cellular tumor antigen p53	98
3	IL-6	Interleukin-6	94
4	VEGFA	Vascular endothelial growth factor A	93
5	CASP3	Caspase-3	88
6	JUN	Transcription factor AP-1	86
6	TNF	Tumor necrosis factor	86
8	MYC	Myc proto-oncogene protein	85
9	EGFR	Epidermal growth factor receptor	84
10	PTGS2	Prostaglandin G/H synthase 2	83

**Table 5:** Key ingredients obtained from ingredient-target-pathway network.

Molecule ID	Name	Degree ≥ 4	Betweenness centrality	Closeness centrality
MOL000098	Quercetin	11	0.1762	0.5714
MOL000173	Wogonin	8	0.0789	0.5128
MOL002714	Baicalein	6	0.0405	0.4545
MOL001689	Acacetin	4	0.0145	0.4167
MOL002928	Oroxylin A	4	0.0171	0.4167
MOL000358	Beta-sitosterol	4	0.0114	0.3922

**Table 6:** Core targets obtained from ingredient-target-pathway network.

Name	Degree ≥10	Betweenness centrality	Closeness centrality
PTGS2	15	0.2576	0.5063
CASP3	13	0.1119	0.4819
TP53	12	0.1144	0.4706
IL-6	11	0.1265	0.4598
TNF	10	0.1098	0.4494
AKT1	10	0.1007	0.4494

**Table 7:** Binding energy between key ingredients and target proteins.

Key ingredients	Binding energy (kcal/mol)					
	PTGS2	CASP3	TP53	IL-6	TNF	AKT1
Quercetin	−2.25	−1.94	−1.74	−1.60	−1.73	−2.90
Wogonin	−2.88	−2.57	−3.22	−1.72	−3.10	−2.44
Baicalein	−4.78	−2.33	−2.4	−2.16	−3.38	−2.53
Acacetin	−4.66	−2.59	−2.74	−2.46	−2.78	−2.46
Oroxylin A	−2.95	−2.54	−3.09	−2.11	−2.94	−2.51
Beta-sitosterol	−2.34	−2.23	−1.95	−1.49	−2.80	−3.16

## Data Availability

All data are fully available without restriction, and all relevant data are included within the paper.
